# Quantitative EEG (QEEG) Measures Differentiate Parkinson's Disease (PD) Patients from Healthy Controls (HC)

**DOI:** 10.3389/fnagi.2017.00003

**Published:** 2017-01-23

**Authors:** Menorca Chaturvedi, Florian Hatz, Ute Gschwandtner, Jan G. Bogaarts, Antonia Meyer, Peter Fuhr, Volker Roth

**Affiliations:** ^1^Department of Neurology, University Hospital BaselBasel, Switzerland; ^2^Department of Mathematics and Computer Science, University of BaselBasel, Switzerland

**Keywords:** Parkinson's disease, QEEG, cognitive decline, Parkinson's disease dementia, neurodegenerative disorders, machine learning

## Abstract

**Objectives:** To find out which Quantitative EEG (QEEG) parameters could best distinguish patients with Parkinson's disease (PD) with and without Mild Cognitive Impairment from healthy individuals and to find an optimal method for feature selection.

**Background:** Certain QEEG parameters have been seen to be associated with dementia in Parkinson's and Alzheimer's disease. Studies have also shown some parameters to be dependent on the stage of the disease. We wanted to investigate the differences in high-resolution QEEG measures between groups of PD patients and healthy individuals, and come up with a small subset of features that could accurately distinguish between the two groups.

**Methods:** High-resolution 256-channel EEG were recorded in 50 PD patients (age 68.8 ± 7.0 year; female/male 17/33) and 41 healthy controls (age 71.1 ± 7.7 year; female/male 20/22). Data was processed to calculate the relative power in alpha, theta, delta, beta frequency bands across the different regions of the brain. Median, peak frequencies were also obtained and alpha1/theta ratios were calculated. Machine learning methods were applied to the data and compared. Additionally, penalized Logistic regression using LASSO was applied to the data in R and a subset of best-performing features was obtained.

**Results:** Random Forest and LASSO were found to be optimal methods for feature selection. A group of six measures selected by LASSO was seen to have the most effect in differentiating healthy individuals from PD patients. The most important variables were the theta power in temporal left region and the alpha1/theta ratio in the central left region.

**Conclusion:** The penalized regression method applied was helpful in selecting a small group of features from a dataset that had high multicollinearity.

## Introduction

Neurodegenerative disorders may begin at any point during the lifetime of an individual and progress for years or decades before becoming clinically manifest (Savica et al., 2010; Reiman et al., 2012). This poses a major obstacle for research into prevention and delays treatment. Dementia is an emergent problem for aging populations, with the two most prevalent neurodegenerative disorders, Alzheimer's disease (AD) and Parkinson's disease (PD), being two of the leading causes (Walker et al., 2015). Cognitive decline due to neurodegeneration occurs gradually, with an intermediate condition between normal cognition and dementia known as mild cognitive impairment (MCI; Petersen et al., 2014). The progression rate from PD-MCI to PD dementia (PD-D) varies depending on age, disease duration and other factors, but one study found it to be approximately 60% over 4 years (Janvin et al., 2006). Other studies found it to be 45–60% while following up for 4–12 years (Buter et al., 2008) and a 49.28% prevalence rate for dementia over 7 years (Sanyal et al., 2014).

A few studies have shown that quantitative EEG (QEEG) could be useful for early prognosis of dementia (Fonseca et al., 2009; Klassen et al., 2011; Dubbelink et al., 2014; Gu et al., 2016). Some alterations in the electrical activity of the brain have also been found to be prevalent in Parkinson's disease patients without dementia (Berendse and Stam, 2007; Stoffers et al., 2007). Benz et al. (2014) reported significant QEEG differences between patients with AD and PD, observing more pronounced slowing of EEG in patients with PD as compared to the AD group. Having a set of QEEG features that could detect patients in the early stages of Parkinson's disease would be useful in providing treatment and care to the individuals. Schmidt et al. (2013) carried out such a study for Alzheimer's Disease (AD) and investigated alpha/theta spectral ratio as a measure to distinguish healthy individuals from patients with AD. Han et al. (2013) recorded EEG's in Parkinson's disease patients and healthy controls and found an increase of relative powers in the delta, theta bands, while observing a decrease of relative powers in the alpha, beta bands. We have investigated the regional powers in Parkinson's disease patients and healthy controls in order to see if a subset of QEEG features obtained from high-density EEG recordings could accurately distinguish between the two groups. Based on previous studies, we speculated that alpha/theta spectral ratio could be a good feature for discriminating between the diseased and healthy individuals. Our aim was also to find an optimal method for feature selection that could deal with high dimensionality, multicollinearity and avoid the risk of overfitting of the data.

The current study explores the differences in high-resolution QEEG data between PD patients (with and without MCI) and healthy controls (HC) at baseline, using regression and machine learning methods (Petersen et al., 2014).

## Materials and methods

### Subjects

Sixty-eight patients with Parkinson's disease were recruited from the Movement Disorders Clinic of University Hospital of Basel from 2011 to 2015 by advertising in the magazine of the Swiss Parkinson's Disease Association. The patients were diagnosed according to the United Kingdom Parkinson's Disease Brain Bank criteria (Gibb and Lees, 1988). A neuropsychological examination was carried out in all individuals during the recruitment process. Knowledge of the German language was a requirement to be included in the study. Nine patients had to be excluded due to presence of other medical conditions and 1 patient dropped out due to an accident. After processing and visually inspecting the EEG data, 8 patients had to be excluded either due to artifacts present or low voltage signals. A group of 50 PD patients (33 males and 17 females) was selected and compared with an age and education matched group of 41 healthy controls (21 males and 20 females), who were recruited from the Memory Clinic, University Center for Medicine and Aging of Basel and from the University Hospital of Basel. The sample size can detect an effect size of 0.59 with a statistical power of 80% at a 5% significance level.

Mean age of the PD group was 68.8 (±7) years, with an average disease duration of 5.3 (±5.1) years, while that of the healthy group was 71.1 (±7) years. The studies were approved by the local ethics committee (Ethikkommission beider Basel, ref. no: 135/11, 294/13, 260/09). All participants gave their written consent.

### Neuropsychological assessment

A comprehensive battery of neuropsychological tests (Strauss et al., 2016) was applied to test for the following cognitive domains: attention, working memory, executive functions, memory and visuo-spatial functions. The raw scores of the tests were normalized and transformed into adjusted z-scores (Berres et al., 2000) based on the data collected for 604 age-, sex-, and education-matched healthy individuals. The tests were used for thorough examination of patients and diagnosis of MCI according to the criteria published by Litvan et al. (2012). Patients with dementia were excluded for this study and only those with MCI or with normal cognition were included.

### EEG recording and processing

A 256-channel EEG System (Netstation 300, EGI, Inc., Eugene, OR) was used to record 12 min of continuous EEG (eyes closed) for all individuals. The participants were seated on reclining chairs, asked to relax while staying awake and to have minimum of eye as well as body movements. Three minutes of EEG data, with single segments of at least 30 s without artifacts (e.g., eye movements, signs of drowsiness), were selected and down-sampled (500 Hz). Data from 214 electrodes (excluding cheeks, neck electrodes) were filtered (0.5–70 Hz) and an inverse Hanning window was used to stitch together shorter segments. Resulting EEG data were re-referenced to average reference and bad channels were interpolated with the spherical spline method. Additionally, the independent component analysis implementation of EEGLAB (Delorme and Makeig, 2004; “runica” with default settings) was used to remove further artifacts. To obtain the power spectra, Welch's method (Welch, 1967) was applied. Relative power was obtained for five frequency bands: delta (1–4 Hz), theta (4–8 Hz), alpha1 (8–10 Hz), alpha2 (10–13 Hz), and beta (13–30 Hz), by calculating the ratio of the signal power within a frequency band to the total signal power (1–30 Hz). The electrodes were mapped to 10 regions of interest on the scalp, corresponding to the left and right frontal, central, parietal, temporal, and occipital. Median and peak frequencies were also calculated from the occipital region. Compared to classical electrode designs (with typically 21 channels), high density electrode systems allow us to aggregate the signals from nearby locations, which in many cases, leads to significant noise reduction.

A total of 79 different measures were extracted and used for further analysis and feature selection. These included global power for each band, power in every region in all five frequency bands, alpha1/theta ratios for all regions and the median as well as peak frequency measures.

### Statistical analysis

Potential confounding by factors, such as age, sex, and education of the patients was accounted for by calculating linear regression models. The dataset had highly correlated features and the goal was to find out which features were important for classification. For this purpose, a comparison was done between Logistic regression and three machine learning methods including Random Forest (Breiman, 2001; Liaw and Wiener, 2002), Support Vector Machine (SVM) (Chang and Lin, 2011) and J48 Decision Trees (Salzberg, 1994) using the Weka software (Hall et al., 2009), version 3.7. Ten-fold cross-validation was applied to all the methods. A ranking of variables was obtained from Random Forest on the basis of mean decrease in accuracy and Gini coefficients. Machine learning methods have been used in quite a few medical studies for prediction and diagnostic classification (Khodayari-Rostamabad et al., 2013; Singal et al., 2013; Johannesen et al., 2016). Differences can be noted in the way each method works and in the results obtained.

While linear and logistic regression generally require linearly separable data, SVMs can handle data that is not linearly separable, using non-linear kernel functions like Radial Basis Function kernels (Pochet and Suykens, 2006). Decision Trees work by creating a flowchart which consists of “leaf” nodes (representing a classification) and decision nodes (which can have several “branches”). Their hierarchical tree structure makes them easy to understand and interpret. A random forest algorithm makes use of several decision trees that are combined in a “bootstrap aggregation” scheme. Based on random subsets of the data, random forests grow a series of individual trees, and the whole forest of such trees can then be used to identify a set of vital features. Random Forests do not require real-valued features and can handle high dimensional data. However, some bias can be introduced with any of the methods, including Random Forest (Strobl et al., 2007).

Additionally, penalized logistic regression was applied to the data to obtain a subset of features that would not be highly correlated to each other. The least absolute shrinkage and selection operator (LASSO) method has been used in different studies for feature selection and computing risk predictive models (Wu et al., 2009; Fontanarosa and Dai, 2011). In many cases, lasso-penalized models have shown improved prediction accuracy while selecting only a limited number of covariates that are included in the model.

The penalized (Goeman, 2010) package in R (R Core Team, 2015) (version 3.2.1) was used to create a logistic regression model and apply the L1-LASSO (Tibshirani, 1996, 1997) penalty. Tenfold cross validation and optimization was carried out to select the tuning parameter. Cross-validated ROC curves were obtained with the ROCR (Sing et al., 2005) package in R.

## Results

Table 1 shows the characteristics of the PD and HC groups. No significant differences were found in the age, education, sex distribution of the patients in the two groups.

**Table 1:** **Demographic characteristics of PD patients and healthy controls (HC)**.

**Parameters**	**HC (*N* = 41)**	**PD (*N* = 50)**	***p*-value (Wilcoxon)**
Age (years)	70 [53, 83]	69 [55, 84]	0.08
Education (years)	12 [8, 19]	14 [9, 20]	0.052
Males	21	33	
Females	20	17	

*The data shown here are the median values and range for each parameter*.

On comparing Logistic Regression, SVM, Random Forest and J48 decision trees, Random Forest was seen to perform better overall with an area under the curve of 0.8 and accuracy of 0.78. The accuracies and AUC values of all methods can be seen in Table 2.

**Table 2:** **Performance measures evaluated by logistic regression and machine learning methods**.

**Method**	**Accuracy**	**AUC**
Random Forest	0.78	0.8
SVM	0.747	0.73
J48	0.68	0.67
Logistic Regression	0.56	0.63

As Random Forest and LASSO are two methods that give a ranked list for feature selection, we focused on these two methods and investigated the subset of features selected by the methods.

**Figure 1:**
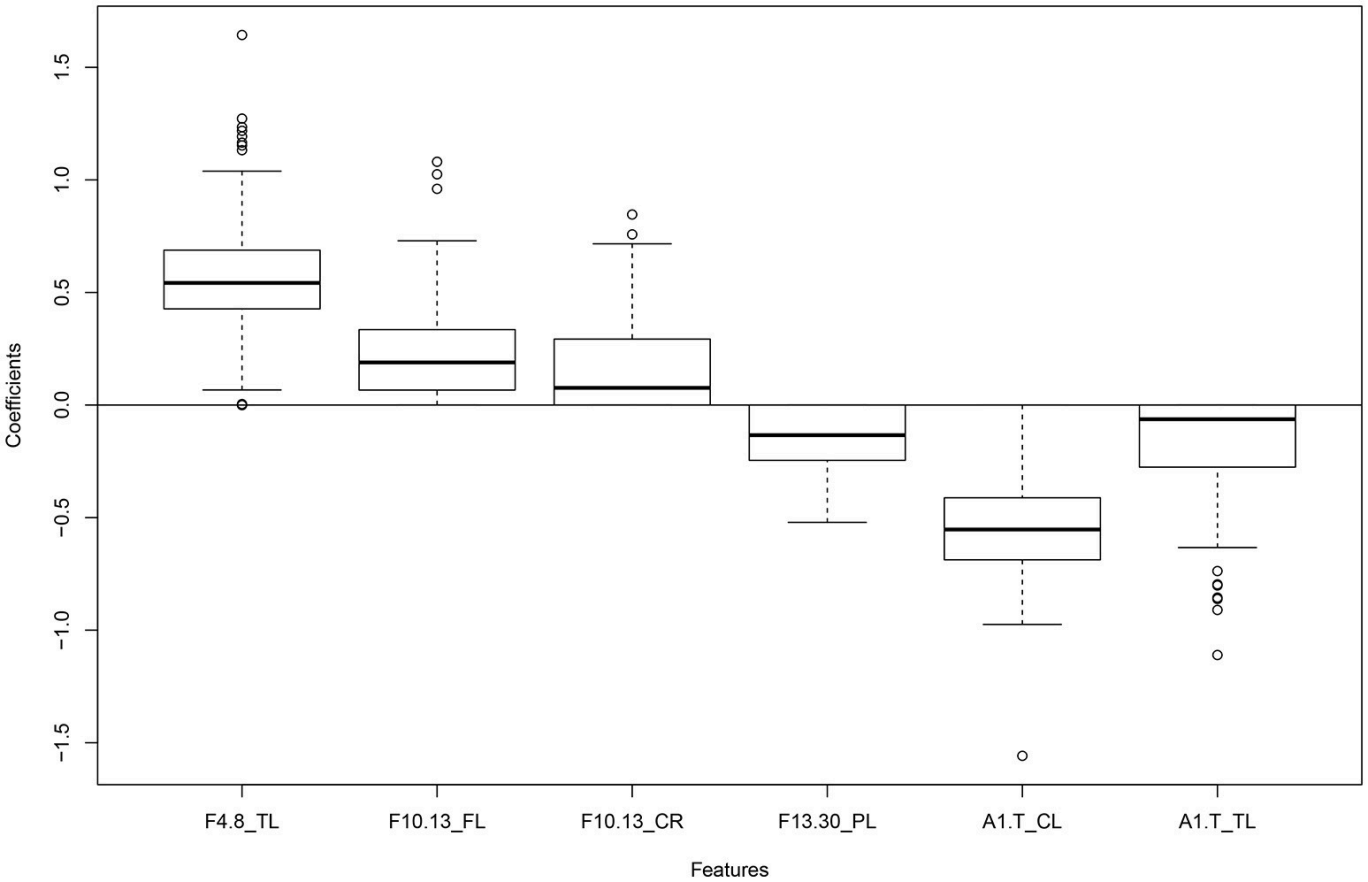
**Box plot showing non-zero coefficients of the penalized logistic regression model obtained after 200 cross validations**.

The penalized logistic regression model obtained from using LASSO revealed the most influential variables in classifying individuals into two groups. Table 3 shows the list of names of the most influential variables. A boxplot depicting the non-zero coefficients of penalized logistic regression model can be seen in Figure 1. The figure shows the coefficients of penalized logistic regression model in which cross-validations were carried out. The median values of the coefficients are seen in the box plot. The different frequency bands are represented as 4.8 (theta), 8.10 (alpha1), 10.13 (alpha2), 8.13 (total alpha), 13.30 (beta). The alpha1/theta ratio is represented as A1.T and the different brain regions are abbreviated as TL/TR (temporal left/right), CL/CR (central left/right), FL/FR (frontal left/right), PL/PR (parietal left/right), CL/CR (central left/right). GP refers to the Global Power in each band).

**Table 3:** **Variables found to be influential in the logistic regression model with LASSO penalty**.

**Variables**	**Coefficients (Median)**
F4.8_TL	0.531
F10.13_FL	0.243
F10.13_CR	0.069
A1.T_CL	−0.586
F13.30_PL	−0.156
A1.T_TL	−0.045

*They are coded as F (Frequency)[Power band in Hertz]_[Brain Region]. Eg: F4.8_TL refers to the theta band power in the temporal left region of the brain. A1.T refers to the alpha1/theta ratio. The median coefficient values depicted correspond to the box plot in Figure 1*.

**Figure 2:**
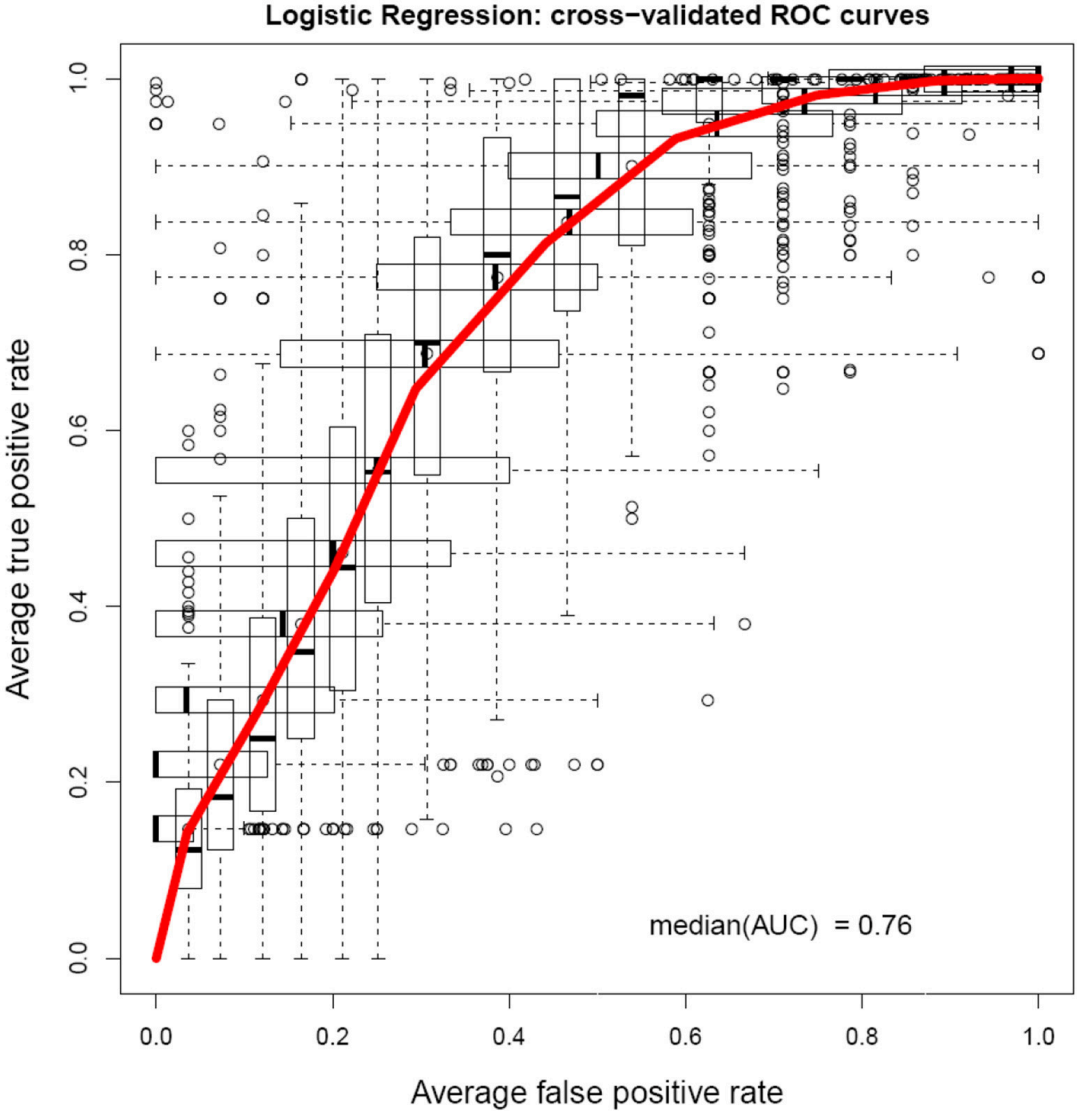
**Cross-validated ROC curve obtained from the logistic regression model shows an AUC value of 0.76**.

A cross-validated ROC curve was plotted after logistic regression is shown in Figure 2. It showed an area under the curve of 0.76.

Alpha1/theta ratio in the central left region and theta power in temporal left were found to be two of the most important features for classification. The average grand spectra for the 10 regions in both groups of individuals can be seen in Figure S1 in the Supplementary section.

**Figure 3:**
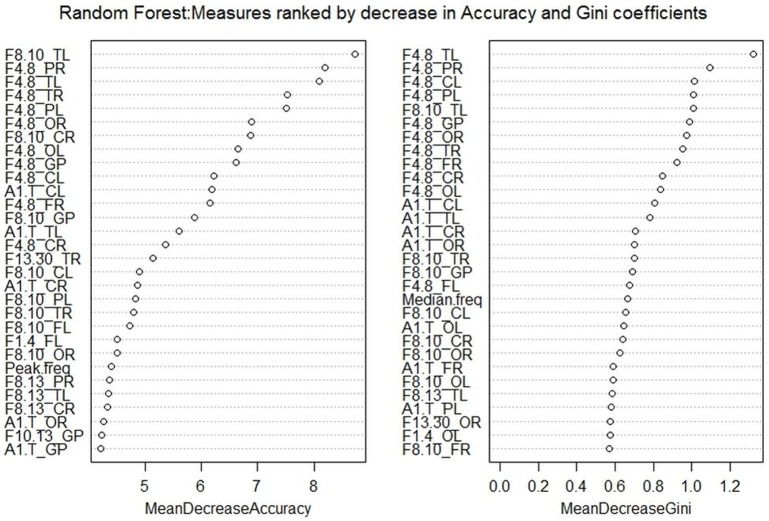
**Variable Importance plots obtained from Random Forest in R show the top QEEG measures ranked on the basis of Mean Decrease in Accuracy and Mean Decrease in Gini coefficients**.

Random Forest ranked the QEEG measures on the basis of a decrease in accuracy of classification and also in decreasing order of the Gini coefficients. A variable is deemed to be more important for the classification of data if its exclusion results in a decrease in the accuracy of the random forest model. This is determined during the out of bag error calculation phase. Hence, the higher the *MeanDecreaseAccuracy* measure for a variable, the greater is its importance. *MeanDecreaseGini* shows how each variable contributes to the homogeneity of nodes in the random forest model. A higher decrease in Gini implies that the variable plays a greater role in the classification process. The top 30 measures obtained from both rankings can be seen in Figure 3.

Both methods selected a few common top features, including theta power in the temporal left region, alpha1/theta ratios in the central left and temporal left regions. The main difference was that LASSO focusses on selecting an optimal set of variables that are not highly correlated to each other but have high accuracy in the prediction model. Random Forest takes the accuracy into account but does not exclude variables that are highly correlated to each other. In this way, a small subset of features for distinguishing the two groups can be obtained using LASSO but a detailed list of influential features can be obtained using Random Forest.

## Discussion

In this study, we investigated 79 frequency measures from 10 regions of interest in groups of PD patients and healthy controls. Our goals were to look for a feature selection method that would solve the problem of multicollinearity, high dimensionality and reduce the risk of overfitting of data. We also wanted to see if alpha/theta spectra ratio would come up as an important feature in distinguishing between diseased and healthy individuals. The penalized logistic regression method (LASSO) applied for classification between the groups resulted in a subset of six measures, reflecting differences in theta, alpha2, beta power, and alpha1/theta ratio in certain regions. Two of the most influential features included theta power in the temporal left region and alpha1/theta ratio in central left region, and were detected by both methods focused on, Random forest and LASSO. As speculated, alpha/theta spectral ratio was seen to be one of the influential features in discriminating between Parkinson's disease patients and healthy individuals.

The regression method with the LASSO penalty has been useful in selecting a group of six features out of seventy-nine. It is good for handling large number of data points and predictors at a time, but can pose a problem if the variables are not relatively scaled. It can be used for different types of data, such as continuous, binomial, etc. However, on carrying out classification with Random Forest, we found that the variables were not ranked in the same way as with LASSO. This could be possibly explained by the fact that a lot of frequency measures, especially in the neighboring regions of the brain, are highly correlated and the LASSO penalty integrated in Logistic Regression only selects one measure out of every group of highly correlated measures.

The final choice of method for feature selection would depend on the question at hand. For obtaining a model that could include a detailed list of the most important variables, Random Forest would be a good choice. If, on the other hand, the goal would be to select a small set of uncorrelated features that could result in comparable prediction accuracy, LASSO would be the preferred method. LASSO selects one set of optimal features for classification, but might not reflect all the features important for clinical diagnosis.

## Author contributions

Contributors MC, FH, and UG carried out data collection. UG, FH, and AM assessed the patients and carried out the neuropsychological testing. MC and FH carried out the data processing; and MC, VR, and JB contributed to the analysis. UG, VR, and PF conceived and designed the study. MC drafted the manuscript and UG, VR, PF, and FH critically revised it.

### Conflict of interest statement

The authors declare that the research was conducted in the absence of any commercial or financial relationships that could be construed as a potential conflict of interest.
