# Supplementary figures and images for: Quantitative EEG (QEEG) Measures Differentiate Parkinson's Disease (PD) Patients from Healthy Controls (HC)
Source: Front Aging Neurosci. 2017 Jan 23;9:3. doi: 10.3389/fnagi.2017.00003 (PMC5253389; doi:10.3389/fnagi.2017.00003)

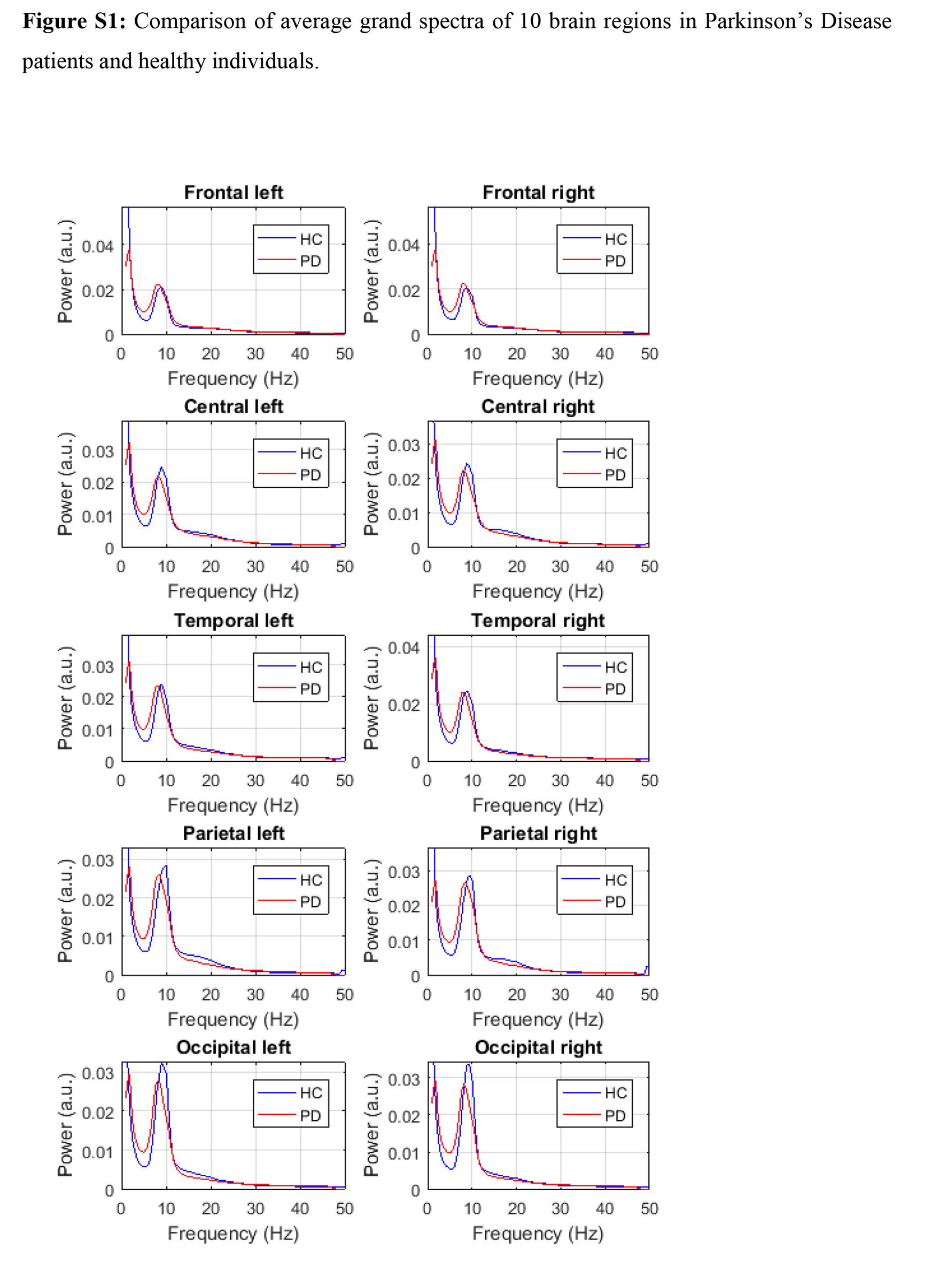

Supplement: Supplementary file 1 [file Image1.TIF]
